# A distinct gastrointestinal symptom profile characterizes clinically relevant fatigue in systemic sclerosis

**DOI:** 10.1007/s10067-026-08361-y

**Published:** 2026-09-08

**Authors:** Francesco Bonomi, Silvia Peretti, Giulia Bandini, Veronica De Silvestri, Cristiano Barbetta, Ilenia Mallia, Khadija El Aoufy, Ginevra Fiori, Serena Guiducci, Alberto Moggi Pignone, Zsuzsanna H. McMahan, Marco Matucci-Cerinic, Cosimo Bruni, Michael Hughes, Silvia Bellando Randone

**Affiliations:** 1 Department of Internal Medicine, University Hospital Careggi, Florence, Italy; 2 Division of Rheumatology, Department of Experimental and Clinical Medicine, University of Florence, Florence, Italy; 3 Department of Internal Medicine, Division of Rheumatology, UTHealth Houston, Houston, TX USA; 4 Unit of Immunology, Rheumatology, Allergy and Rare Diseases (UnIRAR), Inflammation, Fibrosis and Ageing initiative (INFLAGE) IRCCS San Raffaele Scientific Institute, Milan, Italy; 5 Vita-Salute San Raffaele University, Milan, Italy; 6 Inflammation Fibrosis and Ageing Initiative, (INFLAGE), Division of Genetics and Cell Biology, IRCCS San Raffaele Scientific Institute, Milan, Italy; 7 Department of Rheumatology, University Hospital Zurich, University of Zurich, Zurich, Switzerland; 8 Division of Musculoskeletal and Dermatological Sciences, The University of Manchester, Manchester Academic Health Science Centre, Manchester, UK; 9 Department of Rheumatology, Northern Care Alliance NHS Foundation Trust, Salford Care Organisation, Salford, UK; 10 NIHR Manchester Biomedical Research Centre, Manchester University NHS Foundation Trust, Manchester, UK

**Keywords:** Bloating, Fatigue, Gastro-esophageal reflux, Gastro-intestinal, PROMs, Systemic sclerosis

## Abstract

**Objective:**

To identify the specific gastrointestinal (GI) symptoms associated with clinically relevant fatigue in systemic sclerosis (SSc), beyond the established association between overall GI involvement and fatigue.

**Methods:**

We conducted a cross-sectional study including consecutive patients with SSc fulfilling the 2013 ACR/EULAR classification criteria. Fatigue was assessed using the Functional Assessment of Chronic Illness Therapy–Fatigue (FACIT-Fatigue) scale, with clinically relevant fatigue defined as a score ≤ 30. Demographic, clinical, laboratory, and organ involvement data were collected. Individual GI symptom domains (GERD, dysphagia, bloating, diarrhea and constipation), together with the Medsger GI severity score, were analyzed. Bivariate analyses and multivariable logistic regression analyses were performed to evaluate symptom-specific associations with clinically relevant fatigue.

**Results:**

Among 115 patients, clinically relevant fatigue was observed in 42 (36.5%). Patients with FACIT-Fatigue ≤ 30 more frequently reported GERD (90.5% vs 60.3%), dysphagia (40.5% vs 16.4%), bloating (66.7% vs 15.1%), and diarrhea (28.6% vs 11.0%) (all *p* < 0.05). No statistically significant between-group differences were detected in the available objective disease measures. In the primary Firth penalized logistic regression model, bloating (OR 9.11, 95% CI 3.53–25.71) and GERD (OR 4.08, 95% CI 1.29–15.44) were associated with clinically relevant fatigue. Dysphagia showed a directionally consistent but less precise association. In a continuous-outcome sensitivity analysis, GERD, dysphagia, and bloating were associated with lower FACIT-Fatigue scores.

**Conclusions:**

Clinically relevant fatigue in SSc was associated with a distinct profile of patient-reported GI symptoms, particularly bloating and GERD. These findings provide a more granular characterization of fatigue-related GI burden in SSc.

**Table:** 

**Key Points** *• This study moves beyond aggregate gastrointestinal involvement by examining symptom-specific correlates of fatigue in systemic sclerosis.* *• Bloating and GERD were independently associated with FACIT-Fatigue ≤ 30, whereas dysphagia was associated with greater fatigue severity only in the continuous-outcome sensitivity analysis.* *• Clinically relevant fatigue in SSc appears to cluster more closely with patient-reported GI and functional burden than with conventional measures of disease severity.*

## Introduction

Fatigue is one of the most prevalent and disabling symptoms reported by patients with systemic sclerosis (SSc), consistently ranking among the leading determinants of impaired quality of life, reduced functional capacity, and work productivity [1]. Importantly, patients with SSc frequently perceive fatigue as more burdensome than organ-specific manifestations traditionally used to characterize disease severity [2]. In addition, fatigue is increasingly incorporated into composite patient-reported outcome measures (PROMs) and clinical trial endpoints in SSc. Despite this, fatigue remains insufficiently integrated into routine clinical assessment and its clinical meaning in SSc has yet to be fully elucidated.

Fatigue in SSc is widely acknowledged to be multifactorial, reflecting the interplay between organ involvement, pain, functional impairment, psychosocial factors, sleep disturbances, and comorbidities [1–4]. Among these determinants, gastrointestinal (GI) involvement has consistently emerged as one of the strongest disease-related correlates of fatigue in large observational cohorts [5]. However, despite the consistent association observed across cohorts, it remains unclear whether fatigue is linked to the overall burden of GI disease or whether specific GI manifestations account for this relationship.

Importantly, previous large cohort studies have generally treated GI involvement as a single composite construct or binary variable [5]. Consequently, it remains unknown whether the observed association with fatigue reflects the overall burden of GI disease or is driven by specific symptom domains. Distinguishing the contribution of individual manifestations, such as gastroesophageal reflux (GERD), dysphagia, bloating, diarrhea, or constipation, is clinically relevant because these symptoms likely reflect different pathophysiological mechanisms and may have distinct implications for symptom management and future mechanistic studies.

We therefore conducted a cross-sectional study to characterize the GI symptom profile associated with clinically relevant fatigue in SSc. Specifically, rather than evaluating GI involvement as a single aggregate domain, we sought to determine which individual GI manifestations were independently associated with clinically relevant fatigue while also describing the broader clinical and patient-reported phenotype associated with clinically relevant fatigue.

## Materials and methods

### Study design and population

We conducted a cross-sectional, observational study including consecutively recruited patients with SSc attending a tertiary referral center between 01/01/2024 and 31/12/2025. All participants fulfilled the 2013 American College of Rheumatology/European League Against Rheumatism (ACR/EULAR) classification criteria for SSc [6]. Adult patients (≥ 18 years) with available fatigue assessment using the FACIT-Fatigue questionnaire were eligible for this analysis.

Patients with incomplete fatigue assessment were excluded. The study was conducted in accordance with the Declaration of Helsinki and Good Clinical Practice guidelines. The study was approved by the Ethics Committee of Regione Toscana Area Vasta Centro (protocol number 29016/OSS). All participants provided written informed consent prior to inclusion.

### Clinical and demographic data collection

Demographic characteristics (age, sex, body mass index [BMI], smoking status) and disease-related variables were collected from medical records and structured clinical visits. Disease duration was defined as the time from the onset of the first non-Raynaud SSc manifestation.

Clinical variables included SSc subtype (limited cutaneous, diffuse cutaneous, or sine scleroderma) according to the LeRoy classification [7], modified Rodnan Skin Score (mRSS), Raynaud’s phenomenon, digital ulcers, digital pitting scars, calcinosis, telangiectasias, and nailfold videocapillaroscopy pattern (categorized as non-specific, early, active, or late) [8].

Organ involvement was assessed according to standard clinical definitions [9]. Interstitial lung disease (ILD) was defined based on high-resolution computed tomography (HRCT). Pulmonary function tests included forced vital capacity (FVC) and diffusion capacity for carbon monoxide (DLCO). Cardiopulmonary involvement was assessed through pulmonary arterial pressure estimates (sPAP) and left ventricular ejection fraction (LVEF) derived from transthoracic echocardiography, when available.

Comorbidities, including fibromyalgia (defined according to 2016 ACR criteria [10]), sicca syndrome, thyroid disease, and mood disorders, were recorded. The Charlson Comorbidity Index (CCI) is a validated weighted score based on 19 predefined comorbid conditions, each assigned a weight according to disease severity. The total score, obtained by summing individual weights, reflects overall comorbidity burden, with higher values indicating greater disease burden and increased risk of mortality [11, 12].

### GI involvement and symptom assessment

Given the study objective of identifying symptom domains associated with fatigue, at the index clinical assessment, patients were asked whether each GI symptom had been present during the preceding month. Symptoms were recorded dichotomously as present or absent and were not required to be confirmed by objective GI investigations. GERD was defined by troublesome heartburn and/or acid regurgitation, or chronic use of antisecretory therapy [13]; dysphagia as a subjective sensation of difficulty swallowing solids or liquids [14]; bloating as subjective abdominal fullness, pressure, or distension [15]; diarrhea as three or more loose or liquid stools per day, or a clear increase in stool frequency or reduction in consistency compared with usual bowel habits [16]; and constipation as fewer than three bowel movements per week and/or straining or hard stools in at least 25% of defecations, or regular laxative use [17]. Overall, GI disease burden was additionally quantified using the Medsger GI severity score [18], categorized as 0–1 versus ≥ 2 to reflect clinically meaningful involvement, while GI-related symptom burden was evaluated using the UCLA Scleroderma Clinical Trial Consortium Gastrointestinal Tract Instrument (UCLA GIT 2.0), including total score and individual subdomains [19].

### Laboratory assessments

Laboratory variables potentially associated with fatigue were collected, including C-reactive protein (CRP), hemoglobin (Hb) levels, and serum 25-hydroxyvitamin D. These were analyzed using clinically meaningful dichotomous thresholds: CRP elevation (> 1 mg/dL), anemia (Hb < 12 g/dL), and hypovitaminosis D (25-hydroxyvitamin D < 30 ng/mL).

Autoantibody profiles were recorded, including anti-topoisomerase I (anti-Scl-70), anti-centromere, anti-RNA polymerase III, and anti-Ro52.

### PROMs

Fatigue severity was assessed using the Functional Assessment of Chronic Illness Therapy–Fatigue (FACIT-Fatigue) scale, a validated 13-item instrument widely used in systemic autoimmune rheumatic diseases. Scores range from 0 to 52, with lower scores indicating greater fatigue severity. In the absence of an SSc-specific validated threshold, we selected a FACIT-Fatigue score ≤ 30 a priori as a conservative indicator of clinically relevant fatigue based on previously published thresholds in inflammatory rheumatic disease populations [20]. To assess the robustness of the findings to outcome dichotomization, FACIT-Fatigue was also analyzed as a continuous variable in a sensitivity analysis.

Health-related quality of life and symptom burden were evaluated using validated PROMs, including the Health Assessment Questionnaire Disability Index (HAQ) [21], the EULAR Systemic Sclerosis Impact of Disease (Sclero-ID) [22], and the Short Form-36 (SF-36) [23], which assesses physical, mental, and social health domains. In particular, the Sclero-ID questionnaire was analyzed at the level of individual domains rather than as a total score to avoid potential circularity, as the composite score includes a fatigue-related item, which could artificially lead to associations with the study outcome.

All these instruments provided a multidimensional assessment of functional, psychological, and symptom-related burden. Given the conceptual overlap between fatigue and several PROM domains, between-group PROM comparisons were considered descriptive and supportive rather than confirmatory. These measures were not included as covariates in the multivariable models to avoid circularity and potential overadjustment.

### Statistical analysis

Continuous variables were summarized as mean (standard deviation) or median (interquartile range), as appropriate. Categorical variables were reported as counts and percentages based on available observations. Between-group comparisons according to fatigue status (FACIT-Fatigue ≤ 30 vs > 30) were performed using Welch’s *t*-test for approximately normally distributed continuous variables, the Wilcoxon rank-sum test for non-normally distributed or ordinal variables, and Fisher’s exact test for categorical variables. Exact two-sided *p*-values were reported. Effect sizes were expressed as Hedges’ g, rank-biserial correlation, the phi coefficient, or Cramér’s V, as appropriate. Comparisons of demographic and clinical characteristics in Table 1 were considered exploratory and were not adjusted for multiple testing.

**Table 1:** Demographic, clinical, serological, and organ involvement characteristics of systemic sclerosis patients stratified according to the presence of clinically relevant fatigue (FACIT-F ≤ 30). Data are presented as mean (standard deviation), median [interquartile range], or number (percentage), as appropriate. Percentages were calculated using available observations as the denominator. Missing values are reported as the total number of missing observations across both groups. Continuous variables were compared using Welch’s* t*-test or the Wilcoxon rank-sum test, as appropriate, and categorical variables using Fisher’s exact test. Effect sizes are reported as Hedges’ *g*, rank-biserial correlation *r*, the phi coefficient (*φ*), or Cramér’s *V*, as appropriate

Characteristic	FACIT-F > 30 *N* = 73	FACIT-F ≤ 30 *N* = 42	Missing, *n*	Effect size	*p*-value
Demographic and general characteristics					
Age, years, *mean (SD)*	62.8 (11.7)	60.3 (10.5)	0	*g* = –0.22	0.241
Female sex, *n (%)*	54 (74.0)	38 (90.5)	0	*φ* = 0.20	0.051
Tobacco exposure, *n (%)*	26 (35.6)	16 (41.0)	3	*φ* = 0.05	0.683
Charlson Comorbidity Index, *median (IQR)*	3.0 [2.0–4.0]	2.5 [2.0–4.0]	0	*r* = –0.11	0.334
BMI, kg/m^2^, *mean (SD)*	23.6 (4.4)	23.3 (4.4)	10	*g* = –0.06	0.756
Disease duration, years, *mean (SD)*	10.3 (7.1)	10.3 (7.5)	4	*g* = 0.00	0.994
EUSTAR Activity Index, *median (IQR)*	1.0 [0.0–1.2]	1.0 [0.0–1.2]	2	*r* = 0.05	0.631
Serological profile					
ANA positivity, *n (%)*	71 (98.6)	42 (100.0)	1	*φ* = 0.07	> 0.999
Anti-topoisomerase I, *n (%)*	31 (43.1)	12 (28.6)	1	*φ* = 0.14	0.161
Anti-RNA polymerase III, *n (%)*	4 (5.6)	2 (4.8)	1	*φ* = 0.02	> 0.999
Anti-centromere, *n (%)*	33 (45.8)	18 (42.9)	1	*φ* = 0.03	0.846
Anti-Ro52, *n (%)*	12 (16.7)	6 (14.3)	1	*φ* = 0.03	0.796
Cutaneous and vascular manifestations					
SSc subtype, *n (%)*			0	V = 0.10	0.566
* Limited cutaneous SSc*	50 (68.5)	25 (59.5)			
* Diffuse cutaneous SSc*	16 (21.9)	11 (26.2)			
* Sine scleroderma*	7 (9.6)	6 (14.3)			
Raynaud's phenomenon, *n (%)*	71 (97.3)	38 (92.7)	1	*φ* = 0.11	0.349
Current digital ulcers, *n (%)*	6 (8.2)	8 (19.0)	0	*φ* = 0.16	0.136
Puffy fingers, *n (%)*	50 (68.5)	31 (75.6)	1	*φ* = 0.08	0.520
Digital pitting scars, *n (%)*	16 (21.9)	11 (26.8)	1	*φ* = 0.06	0.647
Telangiectasias, *n (%)*	38 (52.8)	27 (65.9)	2	*φ* = 0.13	0.235
Sclerodactyly, *n (%)*	23 (31.5)	10 (23.8)	0	*φ* = 0.08	0.402
Modified Rodnan Skin Score, *median (IQR)*	0.0 [0.0–2.0]	0.0 [0.0–2.0]	4	*r* = –0.06	0.544
Calcinosis, *n (%)*	11 (15.1)	3 (7.3)	1	*φ* = 0.11	0.373
Nailfold videocapillaroscopy pattern, *n (%)*			16	V = 0.20	0.282
*Non-specific*	6 (9.8)	7 (18.4)			
*Early*	20 (32.8)	7 (18.4)			
*Active*	26 (42.6)	20 (52.6)			
*Late*	9 (14.8)	4 (10.5)			
Pruritus, *n (%)*	2 (2.7)	7 (17.5)	2	*φ* = 0.26	0.009
Gastrointestinal manifestations					
GERD, *n (%)*	44 (60.3)	38 (90.5)	0	*φ* = 0.32	< 0.001
Dysphagia, *n (%)*	12 (16.4)	17 (40.5)	0	*φ* = 0.27	0.007
Bloating, *n (%)*	11 (15.1)	28 (66.7)	0	*φ* = 0.52	< 0.001
Diarrhea, *n (%)*	8 (11.0)	12 (28.6)	0	*φ* = 0.22	0.022
Constipation, *n (%)*	16 (21.9)	15 (35.7)	0	*φ* = 0.15	0.129
Medsger GI score ≥ 2, *n (%)*	3 (4.2)	9 (22.5)	4	*φ* = 0.28	0.008
Cardiopulmonary involvement					
Interstitial lung disease on HRCT, *n (%)*	34 (47.2)	20 (47.6)	1	*φ* = 0.00	> 0.999
%FVC, *mean (SD)*	100.5 (20.7)	97.2 (18.6)	6	*g* = − 0.16	0.395
%DLCO, *mean (SD)*	69.7 (17.4)	71.1 (16.0)	6	*g* = 0.08	0.673
Palpitations, *n (%)*	10 (13.9)	10 (23.8)	1	*φ* = 0.13	0.207
%LVEF, *mean (SD)*	57.6 (5.4)	55.5 (6.3)	32	*g* = − 0.37	0.138
Estimated sPAP, *mean (SD)*	25.4 (7.5)	26.1 (5.6)	32	*g* = 0.11	0.609
Musculoskeletal manifestations					
Arthritis, *n (%)*	13 (17.8)	4 (9.8)	1	*φ* = 0.11	0.287
Fibromyalgia, *n (%)*	3 (4.1)	8 (19.5)	1	*φ* = 0.25	0.016
Arthralgia, *n (%)*	22 (30.1)	18 (45.0)	2	*φ* = 0.15	0.150
Comorbidities and laboratory variables					
Sicca syndrome, *n (%)*	19 (26.0)	18 (43.9)	1	*φ* = 0.18	0.062
Thyroid dysfunction (ever), *n (%)*	23 (31.5)	14 (35.0)	2	*φ* = 0.04	0.834
Treated anxiety/depressive disorder, *n (%)*	7 (9.9)	6 (15.0)	4	*φ* = 0.08	0.540
CRP elevation, *n (%)*	7 (9.6)	3 (7.1)	0	*φ* = 0.04	0.744
Anemia, *n (%)*	13 (18.8)	7 (17.9)	7	*φ* = 0.01	> 0.999
Hypovitaminosis D, *n (%)*	25 (48.1)	12 (41.4)	34	*φ* = 0.06	0.645

CRP elevation defined as > 1 mg/dL; anemia defined as hemoglobin < 12 g/dL; hypovitaminosis D defined as serum 25-hydroxyvitamin D < 30 ng/mL

*ANA* antinuclear antibodies, *BMI* body mass index, *CCI* Charlson Comorbidity Index, *CRP* C-reactive protein, *DLCO* diffusing capacity of the lung for carbon monoxide, *FACIT-F* Functional Assessment of Chronic Illness Therapy–Fatigue, *FVC* forced vital capacity, *HRCT* high-resolution computed tomography, *ILD* interstitial lung disease, *LVEF* left ventricular ejection fraction, *mRSS* modified Rodnan Skin Score, *sPAP* pulmonary artery systolic pressure

Because multiple and conceptually overlapping PROM domains were compared between fatigue groups, the Benjamini–Hochberg procedure was applied to PROM comparisons to control the false discovery rate. Both unadjusted and FDR-adjusted *p*-values were reported for these analyses.

The primary multivariable analysis evaluated the association between individual patient-reported GI symptom domains and clinically relevant fatigue, defined as FACIT-Fatigue ≤ 30. Because of the limited number of outcome events and the sparse distribution of some predictors, Firth penalized logistic regression was used to reduce small-sample bias and address potential separation. All five prespecified GI symptom domains (GERD, dysphagia, bloating, diarrhea, and constipation) were entered simultaneously into the primary model. No stepwise or significance-based variable selection procedure was applied. Results were reported as odds ratios with profile penalized-likelihood 95% confidence intervals and corresponding two-sided *p*-values.

To examine the potential effect of residual confounding, an expanded sensitivity model included the same five GI symptom domains together with sex, current digital ulcers, fibromyalgia, and pruritus. These covariates were selected on clinical grounds because of their plausibility as correlates of fatigue. The number of additional covariates was restricted because only 42 fatigue events were available, and the expanded model was therefore considered exploratory.

In a further sensitivity analysis, FACIT-Fatigue was analyzed as a continuous outcome using multivariable linear regression. The model included the five GI symptom domains, sex, current digital ulcers, fibromyalgia, and pruritus. Heteroskedasticity-consistent HC3 standard errors were used, and regression coefficients with 95% confidence intervals were reported. Negative coefficients indicated lower FACIT-Fatigue scores and therefore greater fatigue severity.

Multicollinearity among predictors was assessed using variance inflation factors. For the linear regression model, diagnostic assessment included inspection of fitted values, residuals, studentized residuals, leverage, and Cook’s distance. Potentially influential observations were checked for data-entry errors and retained unless an error was identified. Missing data were reported for each variable, and analyses were conducted using complete-case methodology.

All statistical analyses were performed using R version 4.4.2. A two-sided *p*-value < 0.05 was considered statistically significant.

## Results

### Study population and prevalence of fatigue

A total of 115 patients with SSc were included in the analysis. The mean age was 62.0 ± 11.3 years, and the mean disease duration from the first non-Raynaud manifestation was 10.3 ± 7.3 years. The majority of patients were female (80.0%).

Clinically relevant fatigue (FACIT-F ≤ 30) was reported by 42 patients (36.5%), the remaining 73 patients (63.5%) having no clinically relevant fatigue (FACIT-Fatigue > 30).

### Clinical phenotype associated with clinically relevant fatigue

Demographic and clinical characteristics stratified by fatigue status are summarized in Table 1. Patients with clinically relevant fatigue tended to be more frequently female (90.5% vs 74.0%, *p* = 0.051). No statistically significant between-group differences were detected in age, disease duration, body mass index, smoking status, or Charlson Comorbidity Index.

Patients with clinically relevant fatigue reported a substantially higher prevalence of GI symptoms, including GERD (90.5% vs 60.3%; *p* < 0.001), dysphagia (40.5% vs 16.4%; *p* = 0.007), bloating (66.7% vs 15.1%; *p* < 0.001), and diarrhea (28.6% vs 11.0%; *p* = 0.022). Accordingly, a greater proportion of fatigued patients exhibited more severe GI involvement, as reflected by Medsger GI score ≥ 2 (22.5% vs 4.2%; *p* = 0.008). Notably, associations were not uniform across GI manifestations, as constipation did not differ significantly between groups (35.7% vs 21.9%; *p* = 0.129).

Beyond the GI domain, fatigued patients more frequently reported pruritus (17.5% vs 2.7%; *p* = 0.009) and showed a numerical trend toward a higher prevalence of current digital ulcers (19.0% vs 8.2%, *p* = 0.136). Fibromyalgia was also more prevalent among fatigued patients (19.5% vs 4.1%; *p* = 0.016), whereas arthralgia and arthritis were similarly distributed between groups.

No significant between-group differences were observed in the available measures of skin involvement (mRSS), SSc subtype, autoantibody profile, pulmonary involvement (ILD prevalence, FVC, DLCO), or inflammatory markers.

### PROMs and functional impact

Patients with clinically relevant fatigue reported a substantially greater patient-reported symptom and functional burden across multiple domains (Table 2). Patient-reported GI symptom severity assessed by the UCLA GIT 2.0 was also significantly higher in fatigued patients. Reflux, distension/bloating, diarrhea, social functioning, emotional well-being, and total scores were significantly worse after FDR adjustment (all FDR-adjusted *p* ≤ 0.006), whereas fecal soilage and constipation did not differ between groups.

**Table 2:** Comparison of Patient-Reported Outcome Measures (PROMs) between patients with and without clinically relevant fatigue (FACIT-F ≤ 30). Data are reported as median (interquartile range). *p*-values refer to comparisons between groups

PROM/domain	FACIT-F > 30, median [IQR]	FACIT-F ≤ 30, median [IQR]	Missing, *n*	Effect size, rank-biserial *r*	*p*-value	FDR-adjusted *p*-value
HAQ-DI						
Disability Index	0.00 [0.00–0.50]	0.50 [0.25–1.00]	4	0.496	< 0.001	< 0.001
Sclero-ID						
Raynaud’s phenomenon	3.00 [1.00–5.00]	4.00 [2.00–5.00]	5	0.151	0.191	0.199
Hand function	3.00 [0.75–5.00]	5.00 [3.00–6.00]	5	0.370	0.001	0.002
Upper GI symptoms	1.00 [0.00–4.00]	5.00 [4.00–7.00]	5	0.550	< 0.001	< 0.001
Lower GI symptoms	2.00 [1.00–4.00]	6.00 [2.25–8.00]	5	0.500	< 0.001	< 0.001
Pain	1.00 [0.00–3.25]	5.00 [3.25–7.00]	5	0.652	< 0.001	< 0.001
Life choices	1.00 [0.00–3.00]	5.00 [3.00–7.00]	5	0.560	< 0.001	< 0.001
Body mobility	1.00 [0.00–3.00]	5.00 [3.00–6.00]	5	0.608	< 0.001	< 0.001
Dyspnea	1.00 [0.00–3.00]	3.50 [3.00–6.00]	5	0.519	< 0.001	< 0.001
Digital ulcers	0.00 [0.00–2.00]	0.00 [0.00–4.75]	5	0.182	0.064	0.072
SF-36						
Physical functioning	85.00 [70.00–95.00]	50.00 [35.00–75.00]	13	− 0.596	< 0.001	< 0.001
Role limitations due to physical health	100.00 [25.00–100.00]	0.00 [0.00–50.00]	12	− 0.526	< 0.001	< 0.001
Role limitations due to emotional problems	100.00 [66.70–100.00]	33.30 [0.00–100.00]	12	− 0.454	< 0.001	< 0.001
Energy/fatigue	65.00 [53.75–80.00]	42.50 [25.00–50.00]	1	− 0.680	< 0.001	< 0.001
Emotional well-being	76.00 [64.00–84.00]	54.00 [37.00–68.00]	12	− 0.563	< 0.001	< 0.001
Social functioning	75.00 [62.50–100.00]	50.00 [37.50–62.50]	12	− 0.637	< 0.001	< 0.001
Bodily pain	77.50 [65.00–100.00]	45.00 [22.50–55.00]	12	− 0.700	< 0.001	< 0.001
General health	50.00 [35.00–65.00]	27.50 [15.00–43.75]	12	− 0.527	< 0.001	< 0.001
Health change	50.00 [50.00–50.00]	25.00 [25.00–50.00]	13	− 0.410	< 0.001	< 0.001
UCLA GIT 2.0						
Reflux	0.25 [0.00–0.50]	0.75 [0.35–1.12]	2	0.536	< 0.001	< 0.001
Distension/bloating	0.75 [0.25–1.00]	1.50 [1.00–2.19]	2	0.524	< 0.001	< 0.001
Fecal soilage	0.00 [0.00–0.00]	0.00 [0.00–0.00]	2	0.061	0.425	0.425
Diarrhea	0.00 [0.00–0.50]	0.50 [0.00–1.00]	2	0.291	0.005	0.006
Social functioning	0.00 [0.00–0.33]	0.50 [0.16–1.00]	2	0.494	< 0.001	< 0.001
Emotional well-being	0.00 [0.00–0.28]	0.60 [0.00–1.11]	2	0.444	< 0.001	< 0.001
Total score	0.26 [0.10–0.43]	0.74 [0.43–1.11]	2	0.555	< 0.001	< 0.001
Constipation	0.00 [0.00–0.25]	0.00 [0.00–0.50]	2	0.142	0.161	0.174

*FACIT-F* Functional Assessment of Chronic Illness Therapy–Fatigue, *UCLA GIT 2.0* UCLA Scleroderma Clinical Trial Consortium Gastrointestinal Tract Instrument, *HAQ-DI* Health Assessment Questionnaire—Disability Index, *Sclero-ID* EULAR Systemic Sclerosis Impact of Disease, *SF-36* Short Form 36

HAQ-Disability Index scores were higher in the fatigue group (median 0.50 [IQR 0.25–1.00] vs 0.00 [0.00–0.50]; rank-biserial *r* = 0.496; FDR-adjusted *p* < 0.001). Among Sclero-ID domains, hand function, upper and lower GI symptoms, pain, life choices, body mobility, and dyspnea were all worse in patients with fatigue (all FDR-adjusted *p* ≤ 0.002), with moderate-to-large effect sizes. No significant differences were observed for Raynaud’s phenomenon or digital ulcers after FDR adjustment. As expected, patients with clinically relevant fatigue also had lower scores across all SF-36 domains, including physical functioning, role limitations, emotional well-being, social functioning, bodily pain, general health, and health change (all FDR-adjusted *p* < 0.001). The largest effect sizes were observed for bodily pain (*r* =  − 0.700), energy/fatigue (*r* =  − 0.680), and social functioning (*r* =  − 0.637).

### Multivariable analysis of factors associated with fatigue

**Fig. 1:**
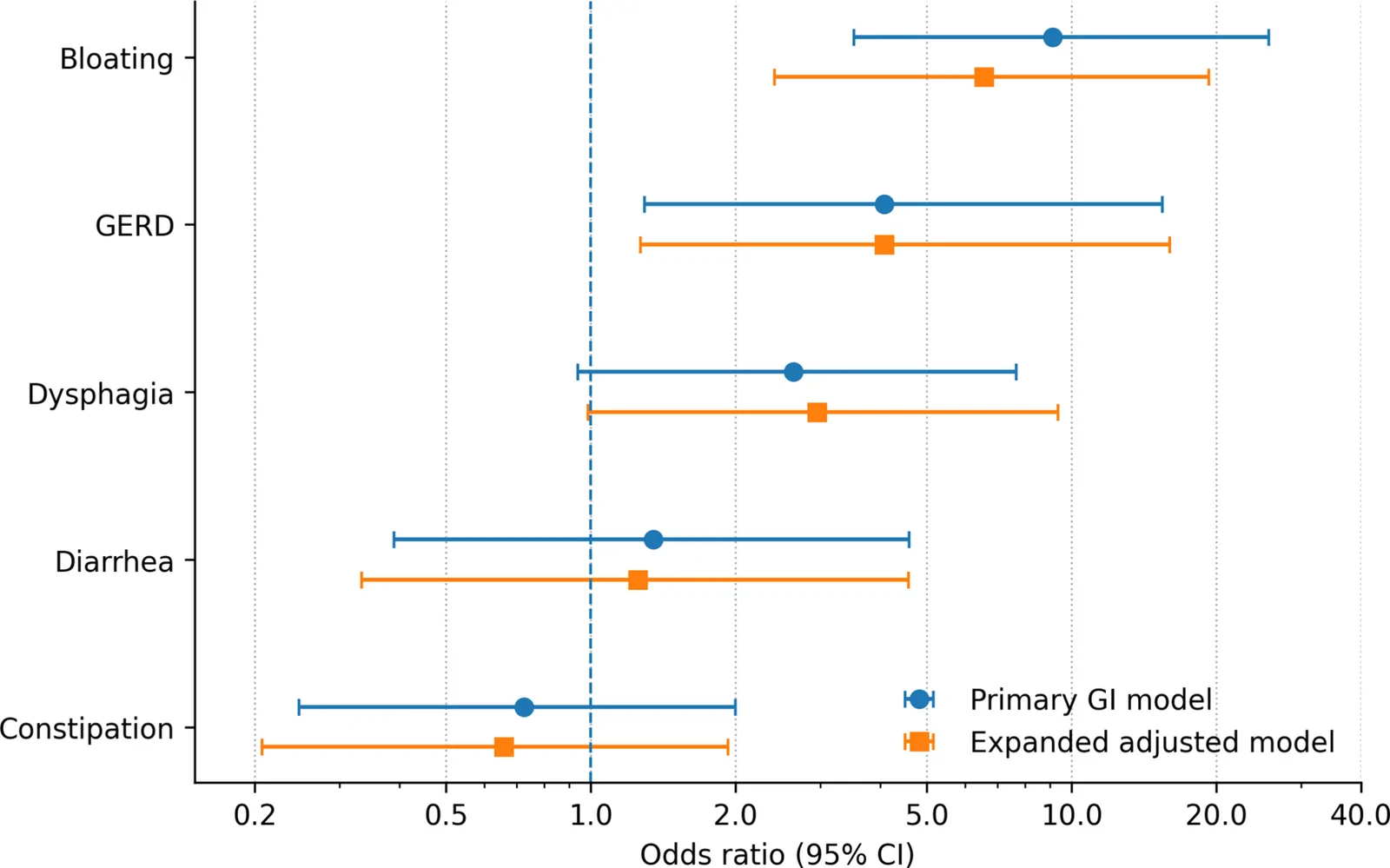
Associations between patient-reported GI symptom domains and clinically relevant fatigue in SSc. Forest plot showing odds ratios and 95% confidence intervals obtained from Firth penalized logistic regression. The primary GI symptom-domain model simultaneously included GERD, dysphagia, bloating, diarrhea, and constipation. The expanded exploratory model additionally adjusted for sex, current digital ulcers, fibromyalgia, and pruritus. Clinically relevant fatigue was defined as a FACIT-Fatigue score ≤ 30. The vertical dashed line indicates an odds ratio of 1.0. GERD, gastroesophageal reflux disease; OR, odds ratio; CI, confidence interval

In the primary Firth penalized logistic regression model, all five patient-reported GI symptom domains were entered simultaneously (Table 3, Fig. 1). Bloating was strongly associated with clinically relevant fatigue (OR 9.11, 95% CI 3.53–25.71; *p* < 0.001), as was GERD (OR 4.08, 95% CI 1.29–15.44; *p* = 0.015). Dysphagia showed an association in the same direction, although it did not reach conventional statistical significance (OR 2.63, 95% CI 0.94–7.66; *p* = 0.066). Diarrhea and constipation were not independently associated with clinically relevant fatigue. The model provided a better fit than the intercept-only model (penalized likelihood-ratio *χ*^2^ = 42.13; *p* < 0.001).

**Table 3:** Firth penalized logistic regression analyses of factors associated with clinically relevant fatigue in SSc. Clinically relevant fatigue was defined as a FACIT-Fatigue score ≤ 30. The primary model included all five prespecified patient-reported gastrointestinal symptom domains. The expanded exploratory model additionally included sex, current digital ulcers, fibromyalgia, and pruritus. Odds ratios and 95% confidence intervals were estimated using Firth penalized logistic regression with profile penalized-likelihood confidence intervals

Predictor	Primary GI symptom-domain model OR (95% CI)	*p*-value	Expanded exploratory model OR (95% CI)	*p*-value
Bloating	9.11 (3.53–25.71)	** < 0.001**	6.56 (2.41–19.32)	** < 0.001**
GERD	4.08 (1.29–15.44)	**0.015**	4.08 (1.27–15.99)	**0.017**
Dysphagia	2.63 (0.94–7.66)	0.066	2.95 (0.99–9.35)	0.053
Diarrhea	1.35 (0.39–4.59)	0.631	1.25 (0.33–4.58)	0.734
Constipation	0.73 (0.25–2.00)	0.539	0.66 (0.21–1.93)	0.451
Male sex	—	—	0.43 (0.10–1.61)	0.216
Fibromyalgia	—	—	2.87 (0.55–18.94)	0.215
Current digital ulcers	—	—	3.64 (0.84–16.87)	0.084
Pruritus	—	—	2.01 (0.37–13.65)	0.429

*GERD* gastroesophageal reflux disease, *OR* odds ratio, *CI* confidence interval

In the expanded exploratory model additionally adjusted for sex, current digital ulcers, fibromyalgia, and pruritus, the associations with bloating (OR 6.56, 95% CI 2.41–19.32; *p* < 0.001) and GERD (OR 4.08, 95% CI 1.27–15.99; *p* = 0.017) remained materially unchanged (Table 3). Dysphagia remained directionally associated with clinically relevant fatigue but narrowly missed statistical significance (OR 2.95, 95% CI 0.99–9.35; *p* = 0.053). Diarrhea, constipation, sex, current digital ulcers, fibromyalgia, and pruritus were not independently associated with the dichotomous outcome. The expanded model also provided a better fit than the intercept-only model (penalized likelihood-ratio *χ*^2^ = 44.69; *p* < 0.001).

In the sensitivity analysis treating FACIT-Fatigue as a continuous outcome, GERD, dysphagia, and bloating were independently associated with lower FACIT-Fatigue scores after adjustment for the other GI symptoms, sex, current digital ulcers, fibromyalgia, and pruritus. GERD was associated with a 5.23-point lower FACIT-Fatigue score (*β* − 5.23, 95% CI − 8.91 to − 1.55; *p* = 0.006), dysphagia with a 6.68-point lower score (β − 6.68, 95% CI − 11.48 to − 1.89; *p* = 0.007), and bloating with a 7.29-point lower score (β − 7.29, 95% CI − 11.70 to − 2.89; *p* = 0.001). Diarrhea and constipation were not associated with FACIT-Fatigue scores. The model included 113 patients and had an adjusted *R*^2^ of 0.34.

No relevant multicollinearity was detected in any of the models. Variance inflation factors ranged from 1.13 to 1.20 in the GI symptom-domain model, from 1.06 to 1.21 in the expanded exploratory model, and from 1.05 to 1.31 in the adjusted continuous-outcome model. Sensitivity analyses excluding potentially influential observations did not materially alter the associations for GERD, dysphagia, and bloating.

## Discussion

In this cross-sectional study, approximately one-third of patients with SSc had clinically relevant fatigue. The principal finding of our study is not simply that GI involvement is associated with fatigue, as this relationship has already been demonstrated in large observational cohorts [5]. Rather, our findings suggest that the association between GI disease and fatigue is not uniform across patient-reported GI symptom domains. Bloating and GERD were independently associated with clinically relevant fatigue, whereas dysphagia showed a directionally consistent but less precise association and was associated with greater fatigue severity in the continuous-outcome analysis. While previous studies consistently identified GI involvement as one of the strongest disease-related correlates of fatigue, they generally evaluated GI disease as a single composite or binary construct. Consequently, it has remained unclear whether fatigue reflects the overall burden of GI involvement or whether particular GI manifestations are primarily responsible for this relationship. By evaluating individual GI symptom domains separately, our study provides a more granular characterization of the GI–fatigue relationship in SSc and helps address an important gap in the current literature.

Our findings extend previous observations from the SPIN cohort and the Australian Scleroderma Cohort, both of which identified GI involvement as an independent determinant of fatigue in SSc [5]. However, these studies did not investigate the relative contribution of individual GI manifestations, precluding a more detailed understanding of which symptoms account for this association. Importantly, our analysis suggests that not all GI symptom domains behaved similarly. While GERD and bloating showed independent associations with fatigue, constipation and diarrhea were not independently associated after simultaneous adjustment for the other GI symptoms. Dysphagia was associated with lower FACIT-Fatigue scores in the continuous-outcome model but did not reach conventional statistical significance in the dichotomous models. These findings suggest that the GI-fatigue relationship is not merely a reflection of cumulative GI symptom burden. Interestingly, despite marked differences in GI symptom burden and patient-reported outcomes, no statistically significant between-group differences were detected in the available measures of skin, serological, pulmonary, and inflammatory disease involvement. This observation suggests that fatigue may cluster more closely with symptom burden than with traditional markers of disease severity.

The associations observed for GERD, dysphagia, and bloating are clinically relevant, although their interpretation requires caution. In particular, the strong association observed for bloating should not be interpreted as evidence of a direct biological relationship. Given the wide confidence intervals and the patient-reported nature of both exposure and outcome measures, bloating may partly capture a broader symptom burden or shared symptom perception rather than a specific pathogenic mechanism. Furthermore, these manifestations represent distinct symptom domains and may influence patients’ daily experience through different pathways. GERD has previously been associated with sleep disturbance in SSc, while dysphagia may affect eating behavior and nutritional intake [24–28]. Bloating may be associated with altered motility or other functional GI disturbances, although no objective assessment of these mechanisms was performed in the present study. Taken together, these symptoms may identify a subgroup of patients with a higher overall patient-reported disease burden, even in the absence of greater involvement according to conventional objective disease measures. Their assessment may therefore help clinicians characterize the symptom profile accompanying fatigue, but the present findings do not establish that treating these manifestations would reduce fatigue.

Although our study was not designed to investigate biological mechanisms, the observed association may also provide a rationale for future investigation of pathways linking GI dysfunction and fatigue, including the hypotheses proposed in other chronic diseases, in which alterations of the gut–brain axis have been implicated in fatigue [29, 30]. The gut–brain axis comprises bidirectional interactions between the GI tract, the immune system, and the central nervous system, and its dysregulation has been associated with persistent fatigue through mechanisms including gut dysbiosis, altered intestinal permeability, and immune activation [31, 32]. However, these pathways were not assessed in our study and therefore should be considered hypothesis-generating. Future longitudinal studies integrating symptom assessment with objective measures of GI function will be required to determine whether these mechanisms contribute to fatigue in SSc.

Conversely, inflammatory markers, cutaneous involvement, and cardiopulmonary parameters did not differ significantly between fatigued and non-fatigued patients. The lack of association with cardiopulmonary variables may reflect the overall preservation of cardiopulmonary function in our cohort, with normal mean FVC, relatively preserved DLCO, and no evidence of relevant cardiac dysfunction on echocardiography despite the presence of ILD in nearly half of the patients. Therefore, fatigue in patients with SSc is likely to be multifactorial, involving biologically plausible mechanisms and psychosocial factors, and extending beyond the simple balance between energy intake and expenditure [33]. Fibromyalgia was more frequent among patients with clinically relevant fatigue, although its adjusted estimate was imprecise and did not reach statistical significance in the expanded exploratory model. Given the small number of patients with fibromyalgia, residual confounding by generalized symptom amplification cannot be excluded.

An additional finding of our study is the close relationship between fatigue and multiple patient-reported outcome measures. Patients with clinically relevant fatigue consistently reported worse physical functioning, greater disability, poorer mental health, and higher GI-related symptom burden, while no statistically significant between-group differences were detected in the available objective disease measures. Fatigue has been shown to explain a considerable proportion of variance in social participation and functional outcomes, highlighting its central role in patients’ lived experience of disease [34]. These findings further support the concept that fatigue represents a multidimensional construct that is only partially explained by conventional measures of disease severity. As PROMs are increasingly incorporated into clinical trials and routine clinical care [35], a better understanding of the clinical determinants of fatigue may improve the interpretation of patient-reported outcomes and facilitate a more individualized assessment of disease burden.

Several limitations should be acknowledged. First, the cross-sectional design precludes causal inference and does not allow determination of whether GI symptoms contribute to fatigue or whether more fatigued patients simply perceive and report greater symptom burden. Second, fatigue, GI symptoms, and several PROM domains were assessed using patient-reported measures, introducing the possibility of common-method bias, shared symptom perception, and circularity. The observed associations should therefore not be interpreted as evidence of objective GI dysfunction. Third, GI involvement was assessed using symptom-based measures rather than objective functional investigations, preventing direct assessment of the relationship between objective GI dysfunction and fatigue. Fourth, the relatively modest sample size and limited number of fatigue events reduced statistical precision. Although Firth penalized regression was used to address sparse data and small-sample bias, the expanded multivariable model included several covariates relative to the number of events and should be considered exploratory. In addition, psychological factors, sleep disturbances, physical activity, and nutritional status were not comprehensively evaluated and may also contribute to fatigue severity. Furthermore, substantial missingness affected selected echocardiographic variables and vitamin D measurements, limiting the strength of between-group comparisons and introducing the possibility of missing-data bias. Finally, the FACIT-Fatigue threshold of ≤ 30 has not been specifically validated in SSc. However, sensitivity analyses using the continuous FACIT-Fatigue score yielded directionally consistent findings, reducing concern that the results were solely driven by dichotomization.

In conclusion, our findings suggest that the well-established relationship between GI involvement and fatigue in SSc is not uniform across the GI manifestations but appears to be primarily associated with a distinct profile of patient-reported symptoms, particularly bloating and GERD. Dysphagia was associated with greater fatigue severity in the continuous-outcome sensitivity analysis but did not reach conventional statistical significance in the dichotomous models. By moving beyond the concept of GI involvement as a single composite construct, this study provides a more granular characterization of the GI correlates of fatigue and supports future longitudinal studies incorporating objective assessments of GI dysfunction.
